# Impacts of Thresholds of Gray Value for Cone-Beam Computed Tomography 3D Reconstruction on the Accuracy of Image Matching with Optical Scan

**DOI:** 10.3390/ijerph17176375

**Published:** 2020-09-01

**Authors:** Se-Won Park, Ra Gyoung Yoon, Hyunwoo Lee, Heon-Jin Lee, Yong-Do Choi, Du-Hyeong Lee

**Affiliations:** 1 Department of Prosthodontics, School of Dentistry, Institute for Translational Research in Dentistry, Kyungpook National University, Daegu 41940, Korea; worm82@naver.com (S.-W.P.); yongdochoi@naver.com (Y.-D.C.); 2 Department of Radiology, Nowon Eulji Medical Center, Eulji University, Seoul 01830, Korea; yoonrg@gmail.com; 3 Department of Dental Clinic, National Medical Center, Seoul 04564, Korea; surgeonsd@gmail.com; 4 Department of Microbiology and Immunology, School of Dentistry, Kyungpook National University, Daegu 41940, Korea; heonlee@knu.ac.kr

**Keywords:** cone-beam computed tomography, gray value, 3D reconstruction, accuracy, image registration

## Abstract

In cone-beam computed tomography (CBCT), the minimum threshold of the gray value of segmentation is set to convert the CBCT images to the 3D mesh reconstruction model. This study aimed to assess the accuracy of image registration of optical scans to 3D CBCT reconstructions created by different thresholds of grey values of segmentation in partial edentulous jaw conditions. CBCT of a dentate jaw was reconstructed to 3D mesh models using three different thresholds of gray value (−500, 500, and 1500), and three partially edentulous models with different numbers of remaining teeth (4, 8, and 12) were made from each 3D reconstruction model. To merge CBCT and optical scan data, optical scan images were registered to respective 3D reconstruction CBCT images using a point-based best-fit algorithm. The accuracy of image registration was assessed by measuring the positional deviation between the matched 3D images. The Kruskal–Wallis test and a post hoc Mann–Whitney U test with Bonferroni correction were used to compare the results between groups (α = 0.05). The correlations between the experimental factors were calculated using the two-way analysis of variance test. The positional deviations were lowest with the threshold of 500, followed by the threshold of 1500, and then −500. A significant interaction was found between the threshold of gray values and the number of remaining teeth on the registration accuracy. The most significant deviation was observed in the arch model with four teeth reconstructed with a gray-value threshold of −500. The threshold for the gray value of CBCT segmentation affects the accuracy of image registration of optical scans to the 3D reconstruction model of CBCT. The appropriate gray value that can visualize the anatomical structure should be set, especially when few teeth remain in the dental arch.

## 1. Introduction

With the widespread use of the cone-beam computed tomography (CBCT) in the dental field, three-dimensional (3D) reconstruction images of the jaws and critical anatomical structures were applied in the diagnostic and treatment modalities in oral implantology, maxillofacial surgery, and orthodontics [1,2,3,4]. CBCT uses a cone-shaped X-ray beam and a flat detector providing a high-resolution image with lower radiation doses and cost than those from multi-slice CT [5,6]. The development of digital impression and dental computer-aided design/computer-assisted manufacturing (CAD/CAM) technologies enhanced the accuracy of implant placement and the convenience of the fabrication of surgical guides reducing the manual work [7,8,9,10,11,12,13]. Accordingly, these 3D image reconstructions and computerized manufacturing have accelerated to optimize implant treatments to be more predictable and evidence-based, from both a surgical and a prosthodontic perspective [14].

Image registration in computer-guided surgery superimposes the optical scan of the oral cavity and the corresponding CBCT data with the same coordinate system [15]. Image merging is a prerequisite for accurate virtual surgical planning, because the surface information of teeth and mucosal tissues in the CBCT is replaced with optical scans, to compensate for the inaccuracy of the CBCT data [16]. The method of image registration is selecting evenly-dispersed anatomical landmarks shown in both CBCT and optical scan images, and further automatic alignment is then processed using adjacent image areas with the automatic best-fit algorithm in planning software [17]. To match CBCT and optical scan images, the digital imaging and communications in medicine (DICOM) images are transformed from a 2D image to a 3D mesh model using medical modeling software. In each of the 2D images of DICOM, the teeth and jaw bone are defined radiopaque images, and the amount of anatomical morphology shown in gray-scale values is designated by controlling the minimum threshold of gray value of segmentation in the software [18]. The gray value is a standard index used in CBCT for representing the detected radiation intensity by quantitative measurement of tissue absorptivity as the Hounsfield unit (HU) of multi-slice CT [19]. Through the setting of the minimum threshold of gray value of segmentation, the shape of the 3D reconstruction image is determined because it is created by computing the voxels that have higher gray values than the minimum threshold.

When the matching areas are deficient or different in shape between the two images in the registration process, image matching could either be failed or not precise. Deviations in the image alignment influence errors in implant position through an inaccurate guide template and cause unavoidable critical complications [20,21]. The control of the gray-value threshold is an important parameter in the segmentation process that directly affects the quality of 3D reconstruction. Depending on the threshold of the gray value of segmentation, scattered radiopaque images in CBCT are rendered different in the 3D reconstruction, which could interfere with the point-based automatic best-fit algorithm. However, there has been limited research on the effects of the minimum threshold of the grey value in the 3D reconstruction of CBCT on the accuracy of image registration of optical scan to the 3D reconstruction models. The objective of this study was to assess the accuracy of image registration of the optical scan to 3D CBCT reconstructions with different thresholds of grey values of segmentation in partial edentulous jaw conditions. The null hypothesis was that the threshold of the gray value of segmentation in CBCT and the number of residual teeth would not result in a different image registration accuracy between the optical scan and the CBCT 3D reconstruction model.

## 2. Materials and Methods

**Figure 1:**
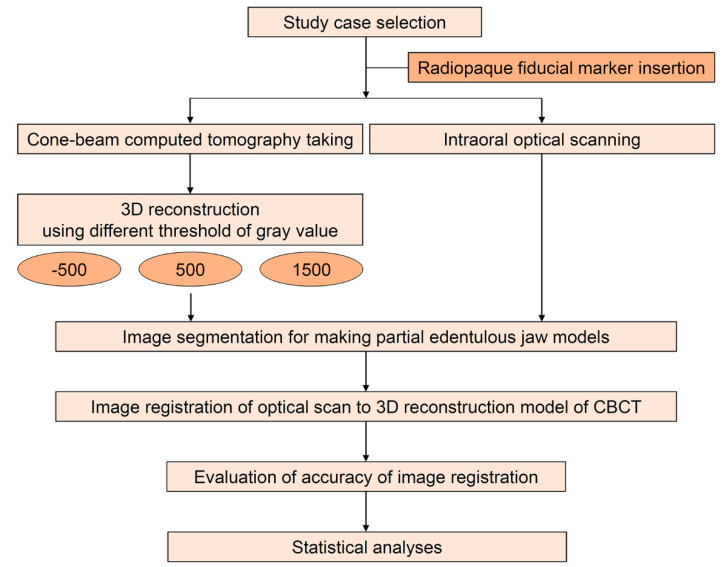
Study workflow.

The workflow of this study is presented in Figure 1. From the patients programmed for orthodontic diagnosis, a dentate case with no metallic prosthesis was selected. The study workflow was approved by the institutional review board of the Kyungpook National University Dental Hospital (No 2020-06-02-00), and written informed consent for the use of imaging data was obtained.

### 2.1. Image Acquisition via Cone-Beam Computed Tomography and Optical Scan

A hemisphere radiopaque fiducial marker with a diameter of 3 mm was made in the posterior area of the hard palate using a light-curing composite resin (CharmFil flow, Denkist, Gunpo, Korea) for supplying a measurement point in this study, and then the image data of the underlying hard tissue and the surface of the oral cavity were obtained. The radiographic data were acquired using a CBCT scanner (Pax-i3D smart; Vatech, Hawseong, Korea) with a field of view of 100 × 80 mm, 0.2 mm voxel size, 85 kVp, 8 mA, and 24 s pulsed scan, and the image slices were saved in the digital imaging and communications in medicine (DICOM) format. The surface data of the oral cavity were digitized using an intraoral optical scanner (CS3600, Carestream, Rochester, NY, USA) and saved in the standard tessellation language (STL).

### 2.2. Image Conversion and Segmentation

**Figure 2:**
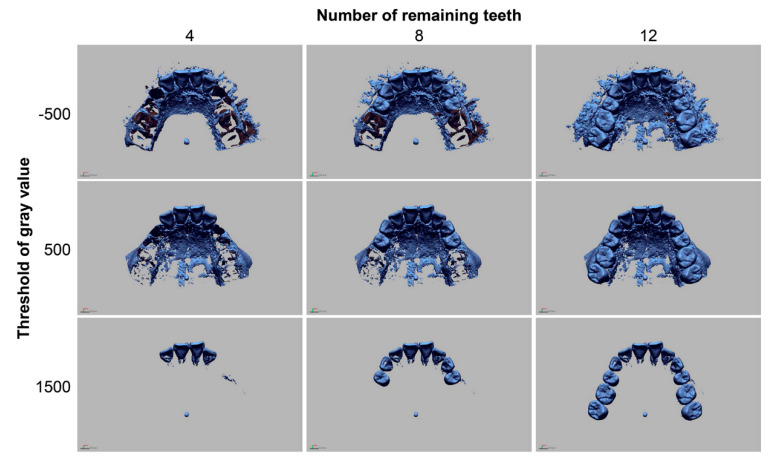
3D reconstruction images of cone-beam computed tomography in the different conditions of the threshold of gray value of segmentation and remaining teeth.

**Figure 3:**
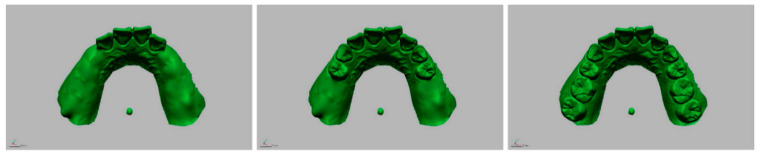
Optical scan images with different numbers of remaining teeth (Left to right: 4, 8, and 12).

The study variables of this experiment were the threshold of gray value that was set in the process of the 3D model reconstruction of radiographic data and the number of remaining teeth. The DICOM data were converted to the 3D mesh models using three different minimum thresholds of gray value (−500, 500, and 1500) in the image-control software (Mimics, Materialise, Leuven, Belgium). Image segmentation of tooth images was followed in the 3D mesh models using image-analysis software (Geomagic DesignX, 3D Systems, Rock Hill, SC, USA) to make models with different numbers of remaining teeth (4, 8, and 12). Accordingly, a total of nine 3D reconstructed models were made according to the radiographic threshold and the number of remaining teeth (Figure 2). The STL image of the surface of the oral cavity was also modified and saved in different files to make scan models with different numbers of remaining teeth (Figure 3).

### 2.3. Image Registration of Optical Scan to 3D Reconstruction Model of CBCT

**Figure 4:**
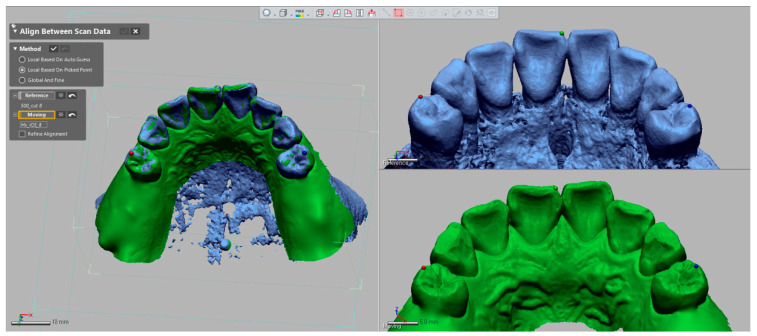
Point-based best-fit image matching between the 3D reconstruction and the optical scan images.

To combine the CBCT-based reconstructed 3D image and the optical scan image, an image-registration process was performed in the image-analysis software (Geomagic DesignX, 3D Systems). In each group, the optical scan images were registered to the corresponding reconstructed 3D images using the point-based best-fit algorithm (Figure 4) [22]. Three matching points in pairs were designated to dental structures discernible in both images with a wide-spread pattern: teeth 11, 12, 22 for the four-teeth condition; teeth 11, 14, 24 for the eight-teeth condition; and teeth 11, 17, 27 for the 12-teeth condition. The anatomic structures, such as the incisal edge or buccal cusp tip of the tooth, were used for reference-matching points. After the point designation, the optical scan images were moved to the closest fit with the corresponding reconstructed images using an iterative closest points (ICP) algorithm in the software programs [17]. The image registration procedure was conducted 5 times for each group by a qualified operator, who had experience in image matching to minimize the human error involved in the selection of matching points. The sample size was determined with power analysis of *F* tests using results of a preliminary test (number of groups = 3, α = 0.05, power = 0.8, effect size f = 0.919, standard deviation = 0.18).

### 2.4. Evaluation of Accuracy of Image Registration

**Figure 5:**
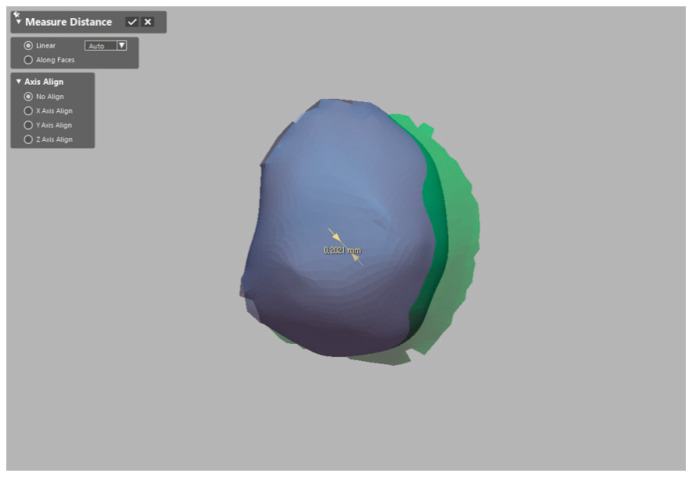
Measurement of 3D linear deviation between the 3D reconstruction and the registered optical scan images using the center point of fiducial marker.

The accuracy of image registration was assessed by means of the positional discrepancy between the 3D reconstructed radiographic image and the registered optical scan image. The positional discrepancy was recorded by measuring the distance between the center points of inserted fiducial markers (3D linear deviation) shown in both images in the image-analysis software (Figure 5). To detect the center point, a virtual circle of a marker outline that was perpendicular to the long axis of the marker was set, and the center point was determined based on the circle. The error measurements were performed by an investigator who was blinded to the purpose of this experiment.

### 2.5. Statistical Analysis

All continuous measurement data were reported as mean ± standard deviation. Statistical analyses were performed using statistical software (IBM SPSS Statistics, v25.0; IBM Corp, Chicago, IL, USA). The Kruskal–Wallis test and the post hoc Mann–Whitney U test with the Bonferroni correction were used to compare the results between groups with a different minimum threshold of gray values and number of remaining teeth. The interaction between the experimental factors on the image registration accuracy was statistically calculated using the two-way analysis of variance (ANOVA). Statistical significance level was set at 0.05. 

## 3. Results

Table 1 shows the image registration error of each condition using the 3D linear deviation values. Of all the thresholds of gray value of segmentation, the registration errors were lowest with the threshold of 500. In the arch image with 4 remaining teeth, the registration error with the threshold setting of −500 was significantly higher than those with the threshold settings of 500 and 1500 (Table 2). In the arch images with 8 and 12 remaining teeth, the registration errors were the lowest with the threshold of 500, followed by those with the thresholds of −500 and then 1500; furthermore, there was a significant difference among the groups in terms of errors.

Different numbers of remaining teeth provided significantly different registration accuracies (Table 3). The registration errors were lowest in the arch image with twelve teeth, followed by in the arch images with eight teeth, and then those with four teeth. The biggest error of 1.89 ± 0.32 mm was observed when the arch image with four teeth was reconstructed with a minimum threshold of −500 gray values, whereas the smallest error of 0.13 ± 0.02 mm was observed when the arch image with the 12-teeth model was reconstructed with a minimum threshold of 500 gray values.

**Figure 6:**
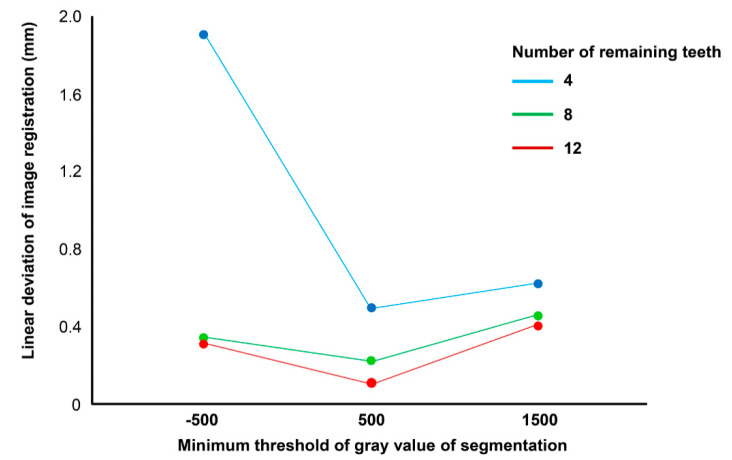
Line graph showing the effect of the threshold of gray value of segmentation on the image registration error of the scan image to cone-beam computed tomography in the dental arch with different numbers of remaining teeth.

The two-way ANOVA test revealed a significant interaction between the minimum threshold of gray value factor and the number of remaining teeth on the registration accuracy (*F* = 53.6, *p* < 0.001; Table 4). Although the trend line of image-registration error had a similar tendency in all arch images having the lowest error values at the threshold of 500, the error was markedly high in the arch image with four remaining teeth and −500 gray-value threshold (Figure 6).

## 4. Discussion

This study was designed to evaluate the impacts of thresholds of gray value of CBCT segmentation on the accuracy of image registration of optical scans on the 3D reconstruction model of the CBCT. For the purpose, the optical scan was registered to 3D reconstruction CBCT models created by different thresholds of gray value of segmentation and number of remaining teeth, and the 3D linear deviations of the registered optical scan were measured. The deviation values were significantly different according to the 3D reconstruction models made by different thresholds of gray value. The deviation values were significantly higher when few teeth remained in the arch in all conditions of thresholds of gray value for CBCT 3D reconstruction. Thus, based on the findings of this study, the proposed null hypothesis, the thresholds of gray value of segmentation in CBCT, and the number of residual teeth would not influence the accuracy of image registration of the optical scan to the CBCT data, was rejected.

The main variables in this study were gray-value thresholds of segmentation and the number of residual teeth in the image registration. The quality of 3D reconstruction image models obtained by CBCT is dependent on acquisition parameters such as tube voltage, tube current, voxel size, the type of the device, the signal-to-noise ratio, the position of the object, the field of view, and anatomic variations [23]. All these factors affect the results of the density of gray-value voxel and further 3D reconstruction. To control these image acquisition factors in the present study, the one set of DICOM data with a fully dentate case was used to create 3D reconstruction models with different thresholds of gray value. Afterward, dental images were selectively removed to make subgroup models with different remaining teeth.

The errors in 3D matching between the reconstructed CBCT image and the optical scan image may be due to discrepancies in shape. In this study, the volume of the 3D reconstruction model was increased with a decrease in the minimum threshold settings. The halation around the mandible can be induced by the scatter and nonlinear partial-volume effect [23,24]. The scattering of photons deteriorates the image, blurring the image borders, and eventually leading to artifacts [25]. Although several strategies have been proposed to reduce the scatter by using physical instruments as anti-scatter grids [26], imaging corrections based on system simulation [27], calibration of the CBCT system [28], and higher scatter levels in CBCT is basically related to the cone-beam projection geometry, that is much higher than in multi-slice CT [29]. Meanwhile, according to the theory of partial-volume effects, the gray value of voxel on the CT image represents the average density value of the corresponding unit when a voxel lies on the borders of two objects of different densities [28]. In larger voxel sizes and lower thresholds for segmentation, the volume of a reconstructed bone could be larger than its real size, and the margin of a reconstructed bone is relatively vague because of the surface-surrounding artifacts [30]. Accordingly, the partial-volume effect can produce deformations in the reconstruction image. Low minimum-threshold settings of gray value visualize more existing artifact images that degrade the quality of CBCT images [25,31]. In the present study, the artifact images were changed to 3D surface polygons in the reconstruction process in the low minimum threshold, causing deformed and irregular surfaces, whereas with increased minimum-threshold settings of segmentation, low gray-density pixels were excluded to be converted to 3D reconstruction images. Some artifacts were hidden, but porous surface images were created in the 3D mesh model, and the loss of surface area needed for the automatic image matching process was also involved. Therefore, an optimal threshold of gray value of segmentation is required to diminish the artifact image involvement and obtain enough area of intact anatomical structure image in 3D reconstruction. 

The use of surface-based image matching is known to be advantageous for computer-guided implant surgery because this workflow is clinically feasible and time-efficient [17,32]. The surface-based matching uses the computer algorithm of iterative closest points (ICPs) that locates paired images in optimal positions by computing the 3D coordinates of the geometrical shape of 3D object surfaces to align images [17,22]. When the image surface point clouds that are used for matching are widely spread in the oral cavity, the matching condition is favorable, and the whole accuracy of image registration can be increased [15,33]. In the present study, a significant correlation was found between the threshold of gray value factor and the number of remaining teeth on the registration accuracy. The deviation error was markedly high in the arch image with four remaining teeth that were created using the −500 threshold of gray value. That is to say, when few teeth remained, low thresholds of gray value of segmentation in CBCT incurred inaccurate image registration of optical scan images. This finding implies that, in the case of extensive tooth loss, particular care should be taken in reducing the creation of adverse artifact images in the 3D reconstruction likely to influence the image matching by not using low thresholds of gray value of segmentation.

The establishment of accurate virtual 3D space visualizing the oro-maxillary anatomical structures brings new diagnostic possibilities, which make the entire treatment procedure more predictable and precise [34,35]. The authors do not know of a previous study that evaluated the relationship between the threshold of gray value of segmentation for CBCT 3D reconstruction and the accuracy of image-matching with an optical scan image. Knowledge of the effects of the image operation factor on the results of image registration can contribute to clinically improve the accuracy of computerized treatments. A limitation of this study was that the radiographic and optical scan data were derived from just one clinical case. The quality of CBCT and optical scan images may vary depending upon the related factors and settings involved. Although this study design was intended to control other confounding factors, multiple and diverse clinical cases should be included in further studies considering the anatomical variations of the dental arch shape, dental morphology, and other influencing factors. More related research on image denoising and artifact suppression methods is required.

## 5. Conclusions

Within the limitations of this study, the accuracy of image registration of optical scan with the 3D reconstruction model of CBCT could be different depending on the threshold of gray value of CBCT segmentation. The accuracy was higher in the case that a high enough number of teeth remained and the set gray value reconstructed the shape of the teeth properly. Particular care should be taken in minimizing the artifact images in the 3D reconstruction when few teeth remain in the dental arch. Further studies are required to confirm the findings of this study in diverse clinical cases and anatomical variations.

## Figures and Tables

**Table 1:** Image registration error (mm) between the optical scan and 3D-reconstructed radiographic images.

N of Teeth	Minimum Threshold of Gray Value of Segmentation			*p*
	−500	500	1500	
4	1.89 ± 0.32	0.52 ± 0.17	0.65 ± 0.10	0.005
8	0.36 ± 0.07	0.21 ± 0.07	0.49 ± 0.05	0.002
12	0.30 ± 0.05	0.13 ± 0.02	0.42 ± 0.03	0.002
*P*	0.006	0.002	0.003	

**Table 2:** Pairwise comparison of registration error between different thresholds of gray value using the post hoc Mann–Whitney U test. * Significant difference (*p* < 0.0,166, Bonferroni correction).

N of Teeth	Threshold	Mean Difference	*p*
4	−500 versus 500	1.37	0.008 *
	500 versus 1500	0.12	0.151
	−500 versus 1500	1.24	0.008 *
8	−500 versus 500	0.15	0.016 *
	500 versus 1500	0.28	0.008 *
	−500 versus 1500	0.13	0.008 *
12	−500 versus 500	0.27	0.008 *
	500 versus 1500	0.29	0.008 *
	−500 versus 1500	0.12	0.008 *

**Table 3:** Pairwise comparison of registration error between different numbers of remaining teeth using the post hoc Mann–Whitney U test. * Significant difference (*p* < 0.0166, Bonferroni correction).

Threshold	N of Teeth	Mean Difference	*p*
−500	4 versus 8	1.53	0.008 *
	8 versus 12	0.06	0.222
	4 versus 12	1.59	0.008 *
500	4 versus 8	0.31	0.008 *
	8 versus 12	0.08	0.008 *
	4 versus 12	0.39	0.008 *
1500	4 versus 8	0.16	0.008 *
	8 versus 12	0.07	0.032
	4 versus 12	0.23	0.008 *

**Table 4:** Variations between the experimental factors in image registration error calculated by two-way analysis of variance.

Source	Sum of Squares	D.F.	Mean Square	*F*	*p*
Threshold	2,395,357.7	2	1,197,678.9	68.6	<0.001
Remaining teeth	4,935,876.4	2	2,467,938.2	141.4	<0.001
Threshold × Remaining teeth	3,740,647.5	4	935,161.9	53.6	<0.001
Error	628,135.2	36	17,448.2		
Total	25,448,153.0	45			
Corrected total	11,700,016.8	44			
